# Embryonic stem cell-derived mesenchymal stem cells alleviate skeletal muscle injury induced by acute compartment syndrome

**DOI:** 10.1186/s13287-022-03000-0

**Published:** 2022-07-15

**Authors:** Xiangkang Jiang, Jingyuan Yang, Fei Liu, Jiawei Tao, Jiefeng Xu, Mao Zhang

**Affiliations:** 1 Department of Emergency Medicine, Second Affiliated Hospital, Zhejiang University School of Medicine, No. 88 Jiefang Road, Hangzhou, 310009 China; 2 Key Laboratory of The Diagnosis and Treatment of Severe Trauma and Burn of Zhejiang Province, Hangzhou, China; 3 Zhejiang Provincial Clinical Research Center for Emergency and Critical Care Medicine, Hangzhou, China

**Keywords:** Acute compartment syndrome, Skeletal muscle injury, Embryonic stem cells, Mesenchymal stem cells, Macrophages

## Abstract

**Background:**

Acute compartment syndrome (ACS), a well-known complication of musculoskeletal injury, results in muscle necrosis and cell death. Embryonic stem cell-derived mesenchymal stem cells (ESC-MSCs) have been shown to be a promising therapy for ACS. However, their effectiveness and potentially protective mechanism remain unknown. The present study was designed to investigate the efficacy and underlying mechanism of ESC-MSCs in ACS-induced skeletal muscle injury.

**Method:**

A total of 168 male Sprague–Dawley (SD) rats underwent 2 h of intracompartmental pressure elevation by saline infusion into the anterior compartment of the left hindlimb to establish the ACS model. ESC-MSCs were differentiated from the human embryonic stem cell (ESC) line H9. A dose of 1.2 × 10^6^ of ESC-MSCs was intravenously injected during fasciotomy. Post-ACS assessments included skeletal edema index, serum indicators, histological analysis, apoptosis, fibrosis, regeneration, and functional recovery of skeletal muscle. Then, fluorescence microscopy was used to observe the distribution of labeled ESC-MSCs in vivo, and western blotting and immunofluorescence analyses were performed to examine macrophages infiltration in skeletal muscle. Finally, we used liposomal clodronate to deplete macrophages and reassess skeletal muscle injury in response to ESC-MSC therapy.

**Result:**

ESC-MSCs significantly reduced systemic inflammatory responses, ACS-induced skeletal muscle edema, and cell apoptosis. In addition, ESC-MSCs inhibited skeletal muscle fibrosis and increased regeneration and functional recovery of skeletal muscle after ACS. The beneficial effects of ESC-MSCs on ACS-induced skeletal muscle injury were accompanied by a decrease in CD86-positive M1 macrophage polarization and an increase in CD206-positive M2 macrophage polarization. After depleting macrophages with liposomal clodronate, the beneficial effects of ESC-MSCs were attenuated.

**Conclusion:**

Our findings suggest that embryonic stem cell-derived mesenchymal stem cells infusion could effectively alleviate ACS-induced skeletal muscle injury, in which the beneficial effects were related to the regulation of macrophages polarization.

## Background

Acute compartment syndrome (ACS), characterized by increasing osseofascial pressure with a severe skeletal muscle injury, is a surgical emergency in clinical practice [1]. Fasciotomy, to fully decompress all compartments, remains the only gold standard treatment for ACS [2, 3]. Despite reperfusion by fasciotomy, paradoxical skeletal muscle injury, which is referred to as ischemia–reperfusion injury (IRI), accompanies ACS. IRI triggers further skeletal muscle damage, which can lead to hypovolemia, rhabdomyolysis, electrolyte and acid–base imbalances, renal failure, and sometimes death [4, 5]. A previous article [6] reported that 44% of ACS patients have long-term limb function defects after fasciotomy. Therefore, it is urgent to develop novel therapeutic methods for ACS.

Experimental evidence suggests that inflammation is one of the main driving factors of secondary injury following ACS [7–9]. Unlike complete ischemia, ACS results in low perfusion of skeletal muscle tissues, which induces early cellular injury and rapid leucocyte activation [9]. The accumulation of activated inflammatory cells adhering to venules directly damages the perfusion of capillaries and increases vascular protein leakage and edema [10, 11]. After fasciotomy, with the increase in tissue perfusion, the inflammatory response and reperfusion-associated skeletal injury further expanded [12]. As ACS-induced skeletal muscle damage is largely irreversible with current treatment approaches, cell-based therapies provide new therapeutic modalities for ACS treatment.

Stem cells, especially mesenchymal stem cells (MSCs), can reduce tissue damage caused by inflammation and oxidative stress while promoting tissue repair after injury.

Previous studies showed that MSCs, derived from bone marrow, placenta, adipose, or other tissues, has been a promising therapy against IRI-related organ injury [13–16]. MSC therapy can reduce neutrophil infiltration in injured skeletal muscle and cell apoptosis caused by inflammation [14], promote skeletal muscle regeneration and functional recovery after injury [13, 16]. However, MSC populations possess heterogeneous biological properties, which could affect their therapeutic efficacy in diseases [17]. Moreover, the numbers of harvested MSCs and their in vitro expansion were a challenge [18, 19]. The successful isolation and culture of embryonic stem cells (ESCs) in vitro [20], which are capable of unlimited self-renewal and differentiation into all cell lineages, opened a new avenue for MSC derivation [21–23].

ESC-derived MSCs (ESC-MSCs) are characterized by a uniform phenotype, relatively stronger proliferation capacity, and stable immunomodulatory properties, and these cells are suitable for large-scale culture. Compared with MSCs from other sources, ESC-MSCs achieve better anti-inflammatory and protective effects in nervous system injuries [24, 25]. These properties and evidence indicate that ESC-MSCs may serve as better candidates for off-the-shelf cell products in future clinical applications than currently available therapies. The efficacy of ESC-MSC infusion in ACS-induced skeletal muscle injury has not been reported, and its potentially protective mechanism requires further investigations.

In this study, we generated MSCs with the same phenotype from embryonic stem cells and investigated their therapeutic potential in ACS rats model. In addition, the molecular basis for their therapeutic effect will be discussed.

## Material and methods

### Animals and ethical statement

All animal experiments were approved by the Institutional Ethics Committee of the Second Affiliated Hospital of Zhejiang University School of Medicine. Male Sprague–Dawley (SD) rats (two months old, 250 ± 10 g) were purchased from Slac Laboratory Animal Co., Ltd. (Shanghai, China) and were housed in a temperature-controlled (22 ± 1 °C) and humidity-controlled (60 ± 5%) room under a 12-h light–dark cycle. The animals had free access to food and water.

### ESC-MSCs preparation and identification

ESC-MSCs were differentiated from ESC using a two-step process. In brief, H9-ESC colonies (YuanSheng Biotech Corporation, Hangzhou, China) were dissociated into small clumps after 3 min of incubation with TrypLE Express and then transferred to ultralow-attachment plates in E8 media (Gibco, Grand Island, NY, USA). After 7 days, embryoid bodies (EBs) were harvested and plated in MSC induction medium composed of Dulbecco’s modified Eagle’s medium (high glucose), 10% fetal bovine serum, and 1 mM l-glutamine. After 2 w, the EB outgrowths were subcultured using TrypLE Express. These cells were ESC-MSCs and were designated passage 0 (P0). The differentiated ESC-MSCs attained a homogenous population of spindle-shaped cells. Passage 4 (P4) ESC-MSCs were used in the following animal experiment.

In this study, phenotypic identification of MSCs was performed using flow cytometry (BD Biosciences, USA) with the following monoclonal antibodies: anti-CD73-PE-Cy7, anti-CD90-APC, anti-CD105-PE, anti-CD14-APC, anti-CD34-PE, anti-CD45-fluorescein isothiocyanate (FITC), CD79a-PE, and HLA-DR-PE. All antibodies were purchased from BD Pharmingen (China). After antibody labeling, data were acquired using an Agilent NovoCyte and analyzed using NovoExpress.

### Establishment of the ACS animal model

The ACS model was established by infusing isotonic normal saline as previously described [8, 9]. In brief, the rats were anesthetized by intraperitoneal administration of 3% pentobarbital sodium. Once the animals were anesthetized, a 24-gauge angiocatheter was inserted into the anterior compartment of the left hindlimb in the experimental group to elevate compartment pressure. The intracompartmental pressure was increased to 80 mmHg and maintained between 80 ± 10 mmHg for 2 h, and a single-incision fasciotomy was performed to decompress the compartment at the end of the experiment. Sham animals underwent all procedures, but the compartment pressure was maintained at the baseline level (0 mmHg). Rats were sacrificed by an intra-arterial overdose (1.5 ml) of 3% pentobarbital sodium, and blood, lung, and tibialis anterior (TA) muscle samples were harvested for further processing.

### Experimental design

**Fig. 1:**
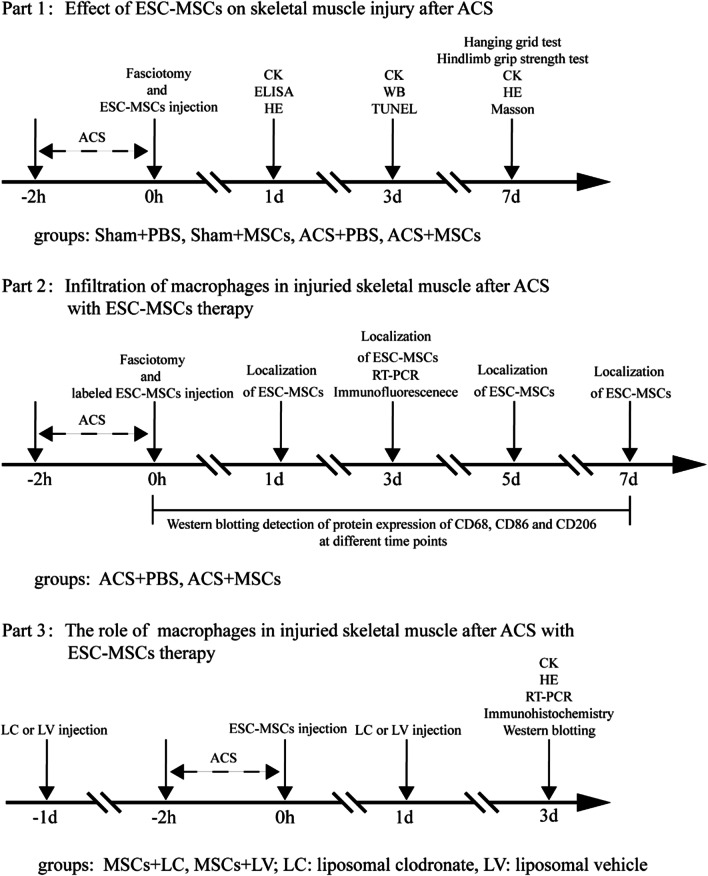
Experimental design and animal groups

This study consisted of three separate parts (Fig. 1). A total of 168 rats were used in this study.

#### Part 1

To investigate the therapeutic effects of ESC-MSCs on ACS-induced skeletal muscle injury, rats were randomly divided into four groups: the Sham + PBS, Sham + MSCs, ACS + PBS, and ACS + MSCs groups at different time points (1 day, 3 days, and 7 days). After fasciotomy, ESC-MSCs (1.2 × 10^6^ in 0.5 ml of PBS) or an equal volume of PBS was administered to the rats by the dorsal penis vein. The muscle edema index and serum creatine kinase (CK) level were measured at 1 day, 3 days, and 7 days after ACS. Moreover, enzyme-linked immunosorbent assay (ELISA) and hematoxylin and eosin (H&E) staining were performed 1 day after ACS. Terminal dUTP nick end labeling (TUNEL) staining and western blotting were performed 3 days after ACS. Additionally, rat ethology was tested 7 days after model induction, and H&E and Masson trichrome staining of the TA muscle was performed 7 days after ACS.

#### Part 2

To clarify whether macrophages polarization was involved in ESC-MSC therapy, rats were randomly assigned to two groups: the ACS + PBS and ACS + MSCs groups at different time points (0 h, 1 day, 3 days, 5 days, and 7 days). Cells were labeled with Paul Karl Horan fluorescent dye (PKH26) before being intravenously injected to track the distribution of ESC-MSCs in vivo. Western blotting and immunofluorescence analysis was performed at 0 days, 1 day, 3 days, 5 days, and 7 days after ACS. Moreover, quantitative real-time PCR was performed 3 days after ACS.

#### Part 3

To identify the role of macrophages in ESC-MSC therapy, macrophages were depleted in rats by intravenous injection of liposomal clodronate (LC). Sham rats were injected with the same amount of liposomal vehicle (LV). Serum CK analysis, H&E staining, quantitative real-time PCR, immunohistochemistry, and western blotting were performed 3 days after macrophage depletion and ESC-MSC therapy.

### ESC-MSCs labeling

For ESC-MSC labeling, we incubated the cells with PKH26 (10 μM) (Sigma, St Louis, MO, USA) at 37 °C for 5 min. After the cells were washed with PBS three times, ESC-MSCs were diluted to a concentration of 2.4 × 10^6^ cells/ml in PBS for injection.

### Macrophages depletion

For the macrophages depletion studies, rats were intravenously injected with 1 ml of (5 mg/ml) LC (Vrije Universiteit, Amsterdam, Netherlands) 1 day prior to and 1 day following ACS injury as previously described [26]. Rats injected with LV (Vrije Universiteit) were used as the control.

### Blood sampling and serum analysis

Blood samples were collected from the abdominal aorta, and serum was obtained by centrifugation. Serum CK levels were measured using a commercial CK kit (Jiancheng Bioengineering Institute, Nanjing, China). The concentrations of tumor necrosis factor-alpha (TNF-α), interleukin 6 (IL-6) and interleukin 10 (IL-10) were measured using ELISA kits (Elabscience, Wuhan, China). The optical density (OD) was measured at 450 nm using a microplate reader (Thermo Fisher Scientific, MA, USA).

### Muscle edema index

The left and right TA muscles were harvested and weighed immediately, and the left/right TA muscle weight ratio was calculated to determine the muscle edema index.

### Rat ethology

The hanging grid test and grip strength test were used to assess skeletal muscle function. The hanging grid test was performed as described previously for mice [27]. The rats were placed individually at the center of a wire mesh screen (2 mm wire thickness). The screen was suspended 50 cm above a plastic cage filled with sawdust bedding, and the grid was inverted with the head declining first. The hanging duration was recorded in three independent trials conducted at least 20 min apart. The data from all three trials were averaged.

The grip strength test was performed using a grip strength meter (Handpi HP-5N, Shenzhen, China) as previously described [28]. The rats were held by the tail and approached the grid slowly until the rat's hind claws grasp the grid, and were gently pulled by the tail until they released their grip. The forces of three trials were recorded and averaged.

### Histological analysis

The TA muscles were fixed in 10% formalin for 24 h and underwent routine dehydration and paraffin embedding. Tissue sections (4 µm) were stained with H&E or Masson trichrome and examined under a light microscope (Leica, Germany).

H&E staining was performed to evaluate the pathological damage to skeletal muscle at 1 day and skeletal muscle regeneration at 7 days after ACS. The histological damage score was determined using 5 random fields selected from each sample at an objective magnification of 20× as previously described [29]. Briefly, sections from each tissue were scored for tissue damage by two investigators in a blinded fashion, but only the highest score was considered. Disorganization and degeneration of the muscle fibers (0: normal, 1: mild, 2: moderate, 3: severe); and inflammatory cell infiltration (0: normal, 1: mild, 2: moderate, 3: severe). Regenerative myofibers were identified as those containing central nuclei [30]. Under an objective magnification of 20×, five random fields were selected from each sample and used to quantify the total number of regenerative myofibers. To measure the diameters of regenerative myofibers, the minor axis diameters of the regenerative myofibers were measured in each TA muscle as previously described [31].

Masson trichrome staining was performed to measure fibrosis 7 days after ACS. The fibrotic area of skeletal muscle was quantitated using 5 random fields selected from each sample at an objective magnification of 20×. ImageJ software was used to calculate the percentage of the fibrotic area.

Skeletal muscle fiber injury in Part 3 was evaluated 3 days after macrophages depletion and ESC-MSC therapy. The injury score was determined based on a protocol established by McCormack et al. [32]. Four random fields of each H&E-stained section at an objective magnification of 20× were examined, and based on the proportion of injured cells (defined by ragged cellular edges, vacuolation, lymphocyte infiltration, or rhabdomyolysis), a numerical value between 0 and 10 was determined.

### TUNEL and dystrophin staining analysis

Apoptotic nuclei in skeletal muscle were examined using double-fluorescent labeling of TUNEL and dystrophin. TUNEL staining was performed according to the manufacturer’s protocol (Roche Inc., Basel, Switzerland). After TUNEL labeling, tissue sections were incubated with a rabbit anti-dystrophin monoclonal antibody (1:200, Cat. 12715-1-AP, Proteintech) followed by an anti-rabbit IgG cyanin 3 (Cy3) (1:200, Cat. SA00009-2, Proteintech). Sections were incubated with DAPI (Meilunbio, Dalian, China) to stain nuclei and examined under a fluorescence microscope (Olympus, Tokyo, Japan). Photomicrographs were merged and saved by Image-Pro Plus software (Olympus). The numbers of TUNEL- and DAPI-positive nuclei were counted and only labeled nuclei that colocalized with dystrophin staining were counted. The data are expressed as the TUNEL index, which was calculated by counting the number of TUNEL-positive nuclei divided by the total number of nuclei. The TUNEL index for each muscle was calculated from five random, nonoverlapping fields at an objective magnification of 40×.

### Immunofluorescence staining

Immunofluorescence staining was conducted as previously described [33] to detect M1 macrophage (CD86) and M2 macrophage (CD206) infiltration in skeletal muscle. Skeletal muscle sections were subjected to antigen retrieval, and the sections were blocked and labeled overnight at 4 °C with rabbit anti-CD86 (1:200, Cat. 13395-1-AP, Proteintech) and mouse anti-CD206 (1:200, Cat. sc-58986, Santa Cruz). After the sections were washed in PBS three times, FITC- or Cy3-labeled secondary antibodies (1:200, Proteintech) were used for the final immunostaining. Sections that were not incubated with primary antibodies were used as negative controls. The sections were incubated with DAPI (Meilunbio) to stain nuclei and examined under a fluorescence microscope (Olympus).

### Western blotting

Western blotting was used to determine protein expression as previously described [34]. TA muscle tissue was lysed in RIPA lysis buffer (Biosharp, Hefei, China). After centrifugation, soluble proteins were quantified with a BCA kit (Biosharp) and separated by sodium dodecyl sulfate–polyacrylamide gel electrophoresis. The proteins were electrophoresed until sufficiently separated and then transferred to polyvinylidene difluoride (PVDF) membranes. The PVDF membranes were blocked with 5% nonfat dry milk in Tris-buffered saline, and then the PVDF membranes were incubated with the following primary antibodies: rabbit anti-tubulin (1:5000, Cat. #5335S, CST), rabbit anti-cleaved caspase3 (1:1000, Cat. #9664S, CST), rabbit anti-caspase3 (1:1000, Cat. #9662S, CST), rabbit anti-Bax (1:1000, Cat. #14796S, CST), rabbit anti-Bcl-2 (1:1000, Cat. #3498S, CST), rabbit anti-CD86 (1:1000, Proteintech), mouse anti-CD206 (1:1000, Santa Cruz), and rabbit anti-CD68 (1:1000, sc-20060, Santa Cruz) at 4 °C overnight. Then, the PVDF membranes were incubated with corresponding horseradish peroxidase (HRP)-conjugated IgG antibodies at room temperature for 2 h. Bands were visualized using an ECL kit (Millipore, Billerica, MA, USA). The band densities were quantified with ImageJ software (NIH).

### Quantitative real-time PCR

RNA was extracted and analyzed using a previously described method [31]. Total RNA was obtained with TRIzol reagent (Invitrogen, MA. USA) and quantified by a Nanodrop spectrophotometer (Thermo Fisher). RNA was then reverse transcribed by the PrimeScript RT reagent kit (Yeason, Shanghai, China). Then, quantitative real-time PCR was performed with SYBR Mixture (Yeason), specific rat primers and cDNA using the Mx3000P real-time PCR system (Agilent Technologies, USA). β-Actin was used as the internal reference. The sequences of primers were used as follows: rat β-Actin forward: 5′-TGTCACCAACTGGGACGATA-3′, reverse: 5′-GGGGTGTTGAAGGTCTCAAA-3′; rat TNF-α forward: 5′-ATGGGCTCCCTCTCATCAGTTCC-3′, reverse: 5′-GCTCCTCCGCTTGGTGGTTTG-3′; rat IL-6 forward: 5′-ACTTCCAGCCAGTTGCCTTCTTG-3′, reverse: 5′-TGGTCTGTTGTGGGTGGTATCCTC-3′; rat IL-10 forward: 5′-CTGCTCTTACTGGCTGGAGTGAAG-3′, reverse: 5′-TGGGTCTGGCTGACTGGGAAG-3′;

### Immunohistochemistry

Immunohistochemistry was performed as previously described [35]. Skeletal muscle sections were subjected to antigen retrieval, and the sections were blocked and labeled overnight at 4 °C with rabbit anti-CD68 (1:200, Santa Cruz). After being incubated with HRP-conjugated secondary antibodies (Boster, Wuhan. China), the sections were treated with an avidin–biotin–peroxidase conjugate (Boster). The reaction was visualized using diaminobenzidine (DAB) substrate chromogen solution (Vectorlabs, CA, USA) after the tissue was counterstained with hematoxylin.

### Statistical analysis

The data are expressed as the means ± standard deviation (SD). Statistical and graph analyses were performed using GraphPad Prism 8.0 (GraphPad Software, USA). For two-group comparisons, significance was assessed by Student’s t-tests. For multiple group comparisons, significance was assessed by One-way ANOVA with Tukey’s post hoc test, two-factor ANOVAs with Bonferroni pair-wise comparisons. Values of *P* < 0.05 were considered statistically significant.

## Results

### Generation and identification of ESC-MSCs

**Fig. 2:**
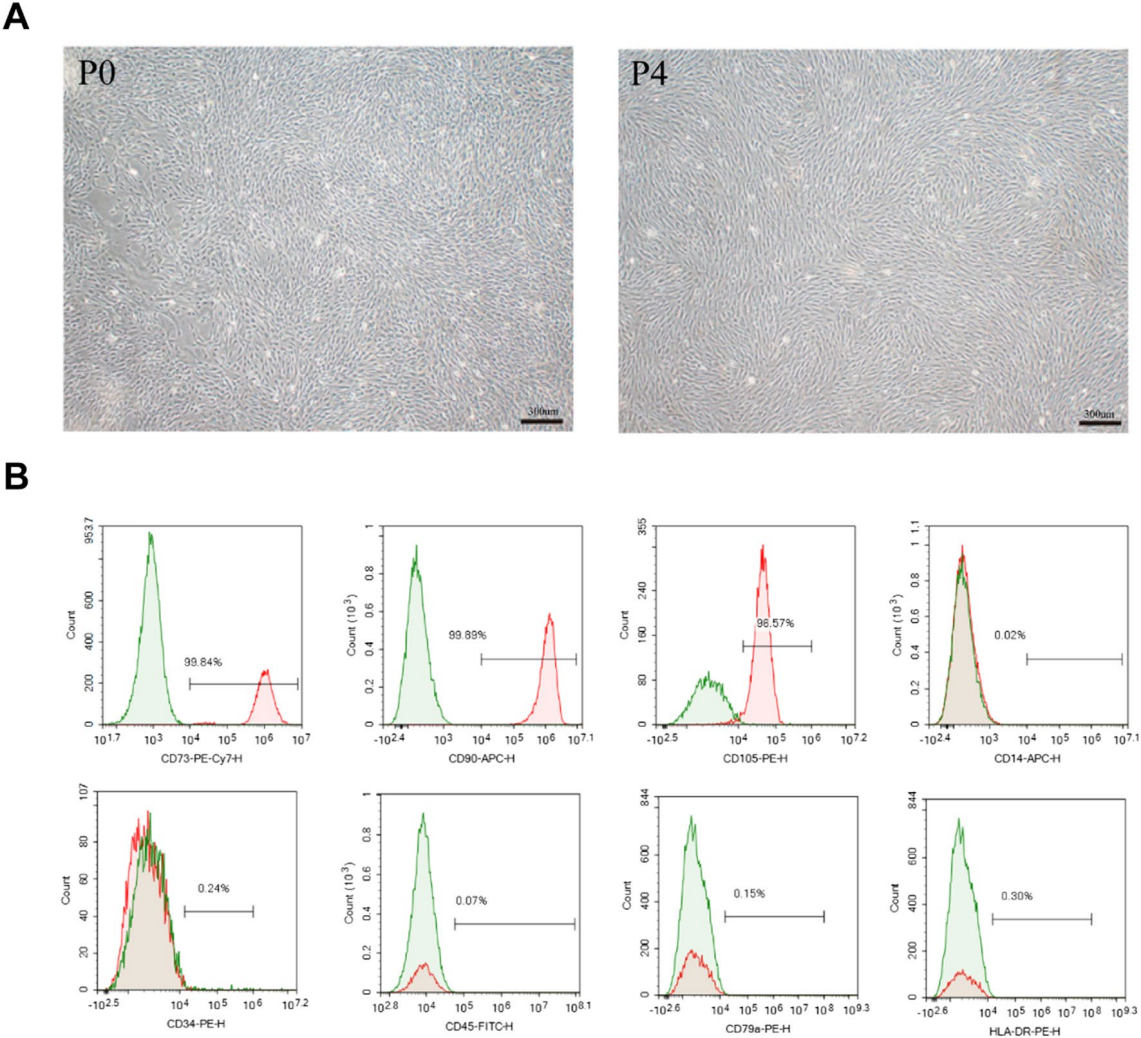
Acquisition and identification of ESC-MSCs. **a** Representative morphology of ESC-MSCs at different stages. **b** The expression of MSC-specific surface markers was analyzed by flow cytometry. Scale bar = 300 μm

Differentiated ESC-MSCs developed a homogenous population of spindle-shaped cells. P4 ESC-MSCs were used in the animal experiment. ESC-MSC morphology at P0 and P4 is shown in Fig. 2a.

The results of ESC-MSC identification are shown in Fig. 2b, and the percentages of CD73-, CD90-, and CD105-positive cells were 99.84%, 99.89%, and 96.57%, respectively, while ESC-MSCs were negative for CD14, CD34, CD45, CD79a, and HLA-DR (all < 0.5%).

### ESC-MSCs reduce the muscle edema index and serum CK levels after ACS

**Fig. 3:**
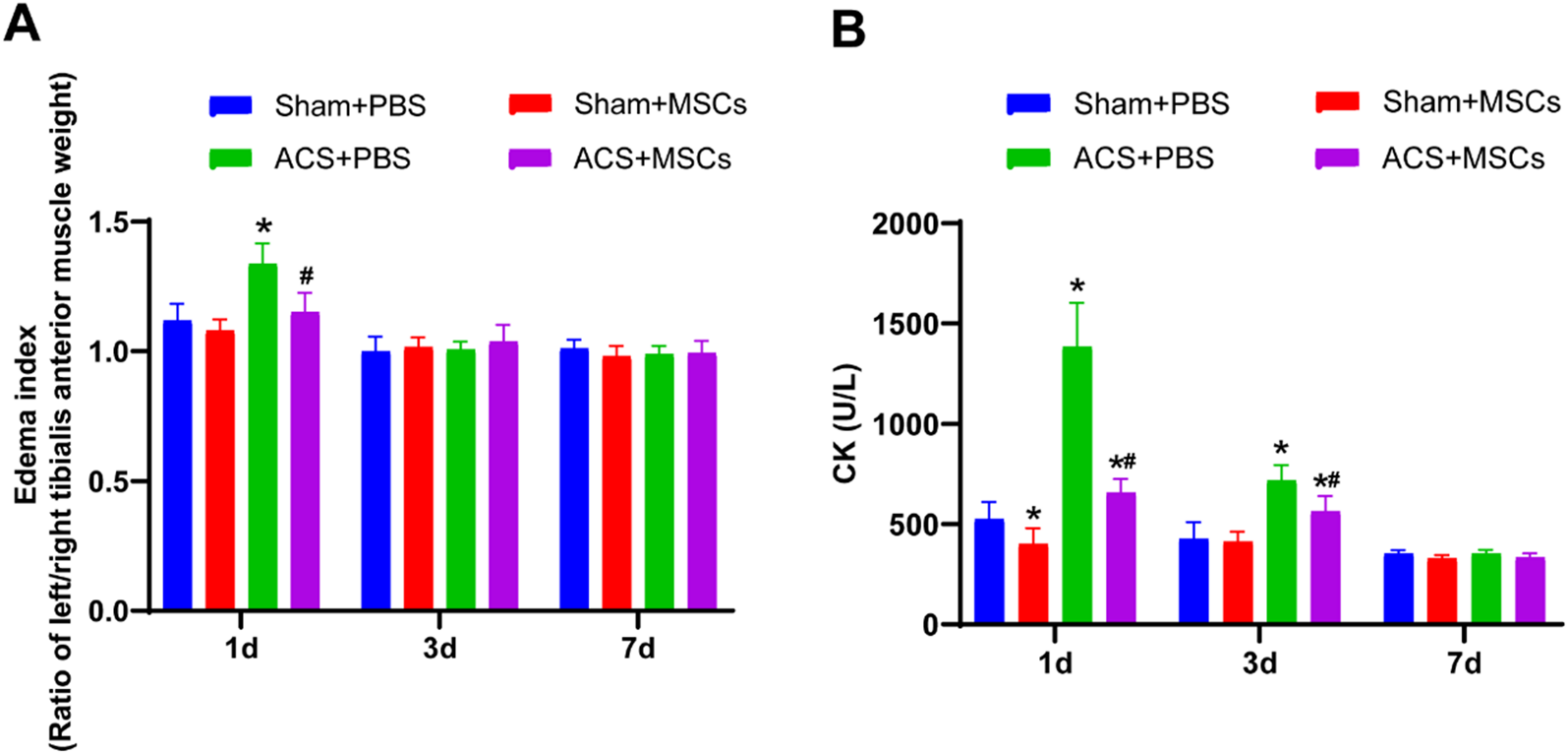
Effects of ESC-MSCs on muscle edema index and serum creatine kinase (CK) levels after ACS. **a** Edema index 1 day, 3 days, and 7 days after ACS. **b** Serum CK levels 1 day, 3 days, and 7 days after ACS. N = 7. The data are presented as the mean ± SD. *P < 0.05 versus sham treated with PBS and ^*#*^*P* < 0.05 versus ACS treated with PBS at the corresponding times

The left/right TA muscle weight ratio was used as an index of edema, and the muscle edema index was similar among the sham groups. ACS injury resulted in a notable increase in the muscle edema index, and ESC-MSCs effectively reduced the muscle edema index 1 day after ACS (Fig. 3a). A similar trend was observed regarding serum CK levels (Fig. 3b). 3 days after ACS, the muscle edema index and serum CK levels gradually returned to normal, and there were no significant differences between the ACS groups and sham groups in the muscle edema index.

### ESC-MSCs alleviate systemic inflammation and histopathological alterations in skeletal muscle after ACS

**Fig. 4:**
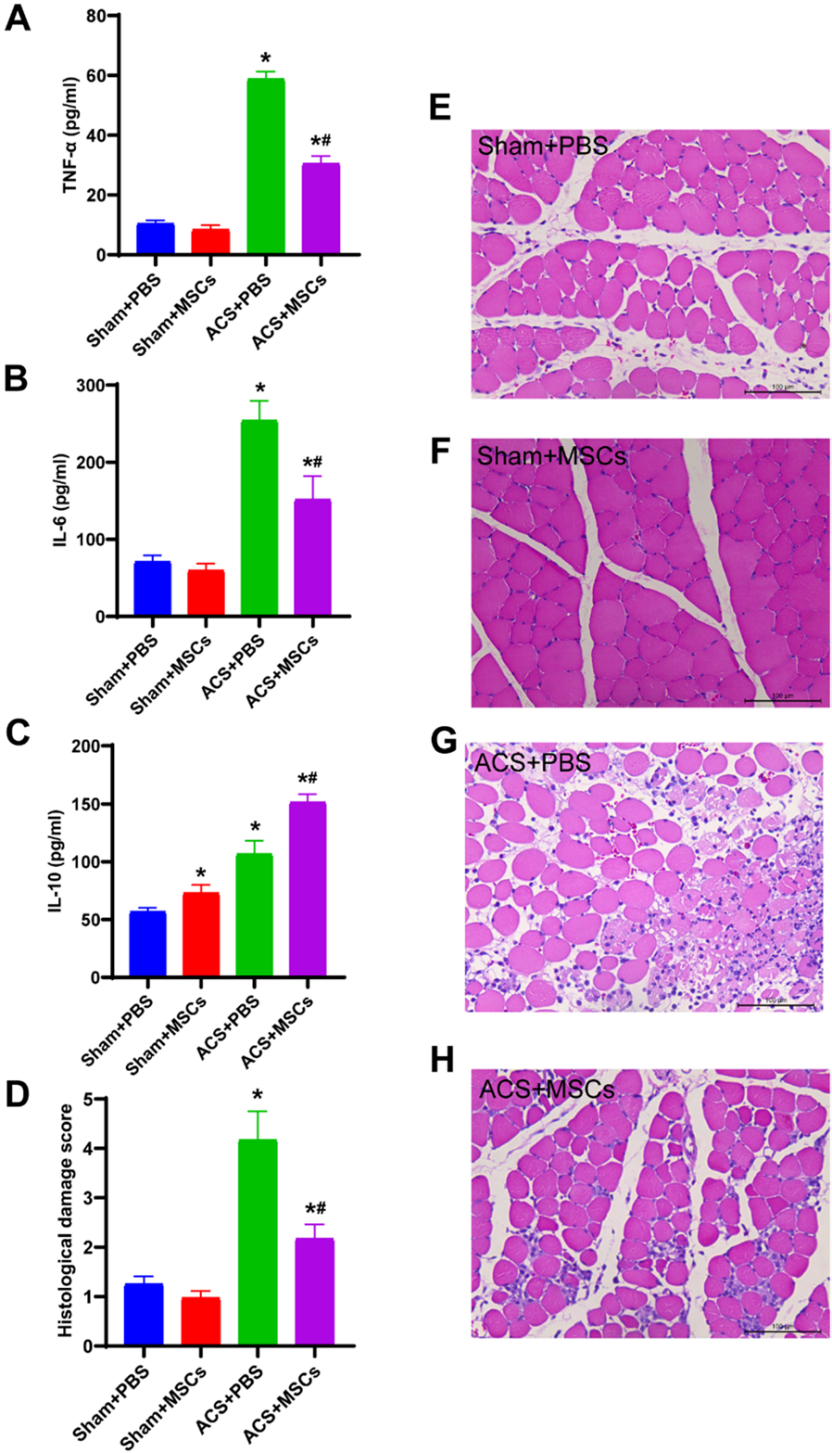
Effect of ESC-MSCs on systemic inflammation and the histopathology of skeletal muscle 1 day after ACS. **a-c** The serum levels of the cytokines TNF-α, IL-6, and IL-10 were measured by ELISA. **d** Skeletal muscle damage scores 1 day after ACS. *N* = 7. **e–h** Representative images of the TA muscle cross-sections in each group (original magnification, ×200). The data are presented as the mean ± SD. *P < 0.05 versus sham treated with PBS and ^*#*^*P* < 0.05 versus ACS treated with PBS

To examine the effect of ESC-MSCs on systemic inflammation and skeletal muscle injury 1 day after ACS, ELISA and histologic examination was performed. The ELISA results showed that ESC-MSCs significantly decreased serum levels of the proinflammatory cytokines TNF-α and IL-6 and increased the anti-inflammatory cytokine IL-10 (Fig. 4a–c). Severely disorganized and degenerated muscle fibers and inflammatory cell infiltration were observed in ACS rats (Fig. 4g). Infusion with ESC-MSCs significantly reduced skeletal muscle fiber disorganization and inflammatory cell infiltration (Fig. 4h) and lowered the histological damage score (Fig. 4d).

### ESC-MSCs decrease skeletal muscle apoptosis after ACS

**Fig. 5:**
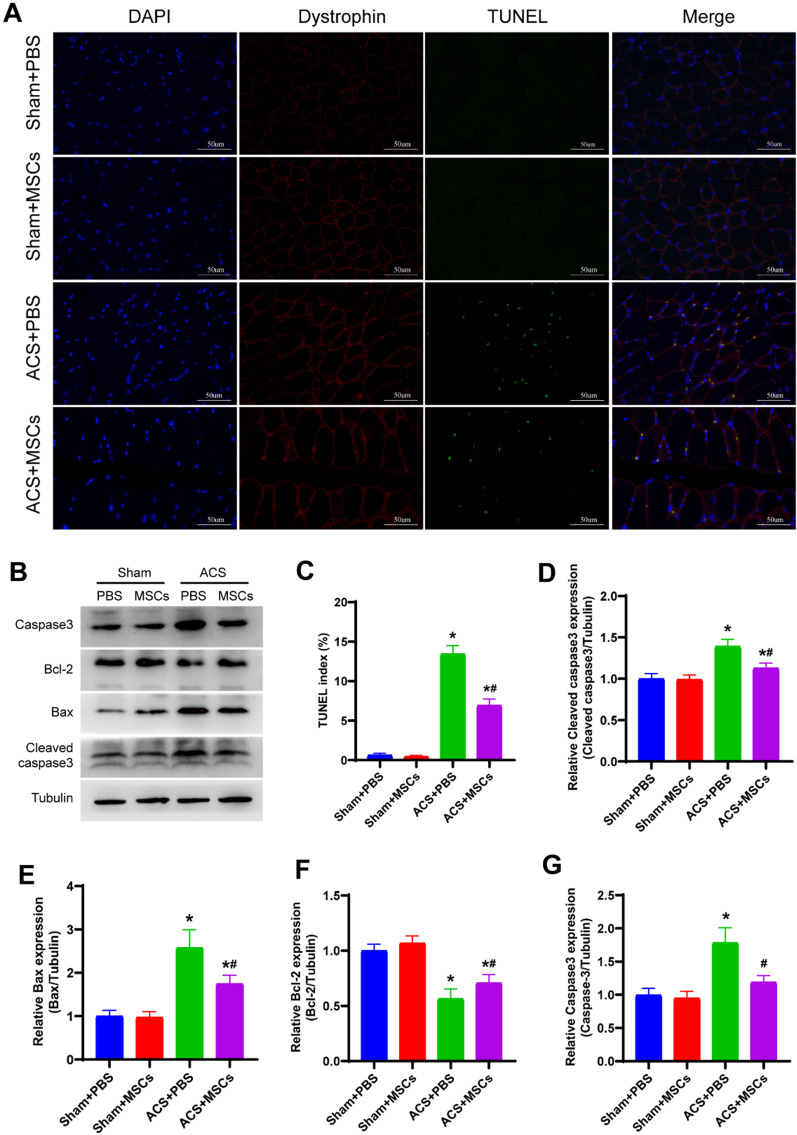
Effect of ESC-MSCs on skeletal muscle apoptosis 3 days after ACS. **a** Representative images of TUNEL and dystrophin staining. **b** Representative western blotting images. **c** Quantitative analysis of TUNEL and dystrophin staining. **d** Quantitative analysis of cleaved caspase3 expression. **e** Quantitative analysis of Bax expression. **f** Quantitative analysis of Bcl-2 expression. **g** Quantitative analysis of caspase3 expression. N = 7. The data are presented as the mean ± SD, and there were no significant differences among the sham groups. *P < 0.05 versus sham treated with PBS and ^*#*^*P* < 0.05 versus ACS treated with PBS

Apoptotic cells were examined using the TUNEL assay. 3 days after ACS, rats in the ACS group exhibited a significant increase in the skeletal muscle apoptotic index, and ESC-MSCs suppressed ACS-induced apoptosis in skeletal muscle cells (Fig. 5a). In addition, western blotting was used to determine the expression of apoptosis-related proteins in skeletal muscle, and the data suggested that the protein levels of cleaved caspase-3, Bax, and Caspase-3 were significantly increased, while the protein level of Bcl-2 was decreased in the ACS group compared to the sham group. Compared with PBS, ESC-MSCs markedly suppressed the protein levels of cleaved caspase-3, Bax, and caspase-3 and promoted the protein level of Bcl-2 (Fig. 5b). These findings suggested that ESC-MSCs suppressed apoptosis in skeletal muscle cells after ACS.

### ESC-MSCs alleviate skeletal muscle fibrosis and promote the regeneration of muscle fibers and muscle function recovery after ACS

**Fig. 6:**
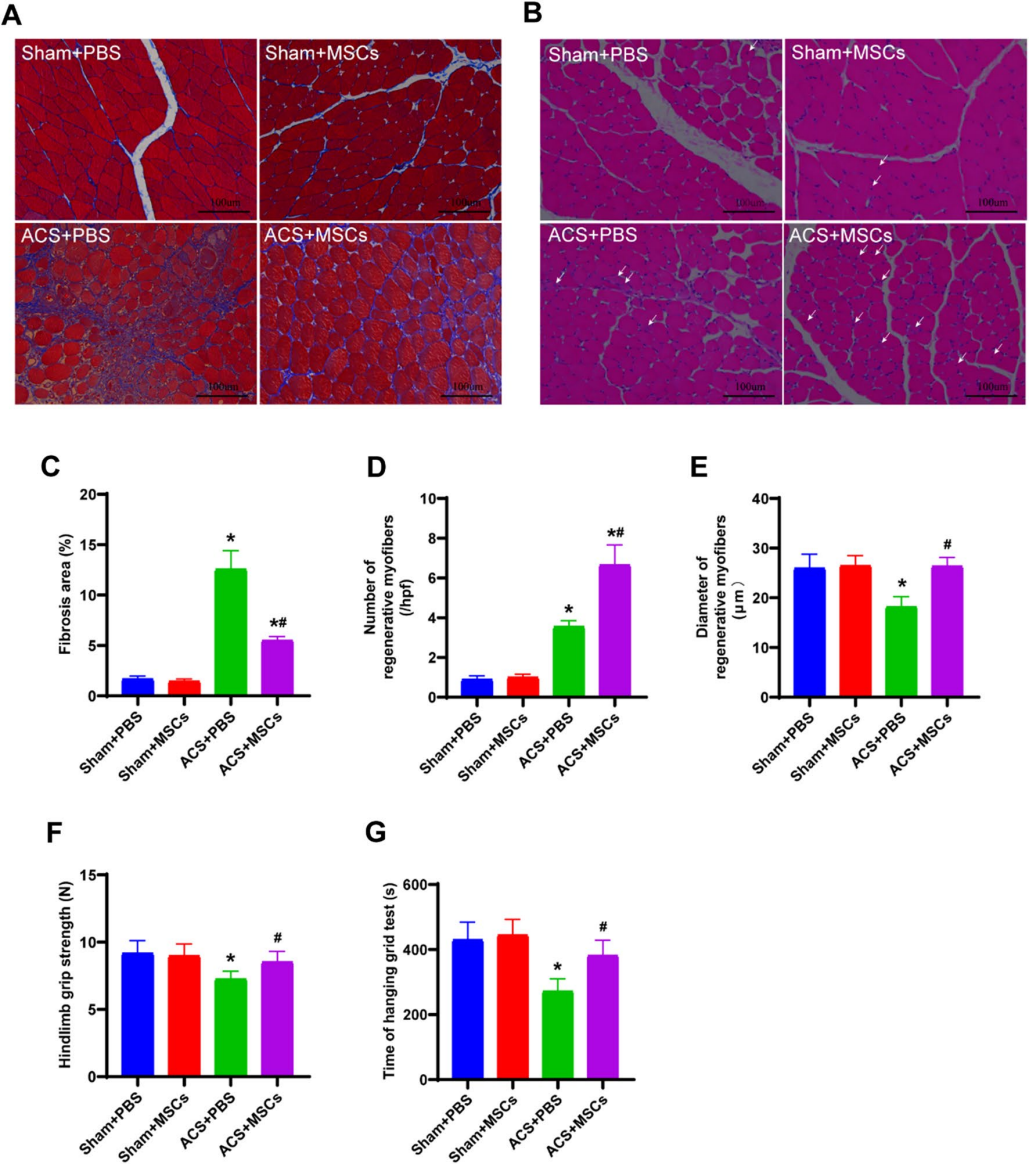
Effect of ESC-MSCs on skeletal muscle fibrosis, regeneration, and functional recovery 7 days after ACS. **a** Representative image of fibrosis in each group, as shown by Masson trichrome staining. **b** Representative images of muscle regeneration, as shown by H&E staining. Regenerating myofibers were defined by myofibers containing a central nucleus (original magnification, ×200). **c** Quantitative analysis of the fibrotic area in each group. **d** and **e** Quantitative analysis of the number (**d**) and diameter (**e**) of regenerating myofibers in each group. **f** and **g** The grip strength (**f**) and hanging time (**g**) in each group. *N* = 7. The data are presented as the mean ± SD, and there were no significant among the sham groups. *P < 0.05 versus sham treated with PBS and ^*#*^*P* < 0.05 versus ACS treated with PBS

To investigate skeletal muscle fibrosis and regeneration 7 days after ACS, we stained TA muscle sections with H&E and Masson trichome. Skeletal muscle fibrosis and regenerated muscle fibers were significantly increased in ACS rats compared to sham rats, and ESC-MSCs effectively reduced skeletal muscle fibrosis after ACS (Fig. 6a) and increased the number and diameter of regenerated muscle fibers (Fig. 6b). In addition, there was also an increase in the hanging grid test time (Fig. 6f) and hindlimb grip strength (Fig. 6g).

### ESC-MSCs regulate macrophages polarization and reduce the inflammatory response in skeletal muscle after ACS

**Fig. 7:**
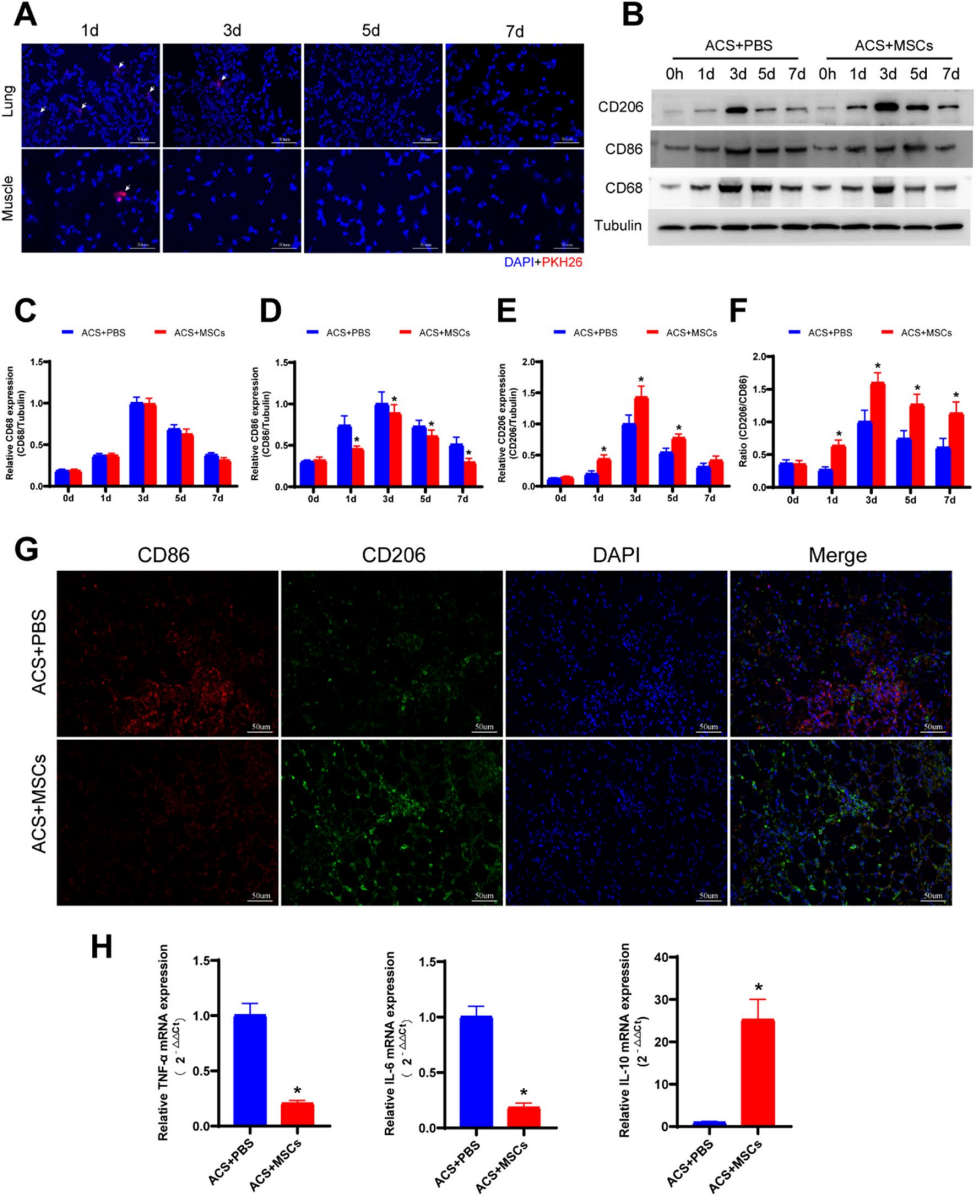
Effects of ESC-MSCs on macrophage polarization and the mRNA expression of inflammatory factors in skeletal muscle after ACS. **a** The distribution of labeled ESC-MSCs in the lung and skeletal muscle tissue of rats at various time points. **b** Representative western blotting images. **c** Quantitative analysis of CD68 expression. **d **Quantitative analysis of CD86 expression. **e** Quantitative analysis of CD206 expression. **f** Quantitative analysis of CD206/CD86 ratio. **g** Representative images of M1-polarized macrophage (CD86) and M2-polarized macrophage (CD206) infiltration in skeletal muscle after PBS or ESC-MSC infusion. **h** Quantitative real-time PCR data showing the mRNA levels of TNF-α, IL-6, and IL-10 in skeletal muscle after PBS or ESC-MSC infusion. *N* = 7. The data are presented as the mean ± SD. *P < 0.05 versus ACS treated with PBS

To track the distribution of ESC-MSCs after intravenous injection, PKH26-labeled ESC-MSCs were infused into rats after fasciotomy, and the distribution was confirmed by examining frozen tissue sections after 1 day, 3 days, 5 days, and 7 days. PKH26-labeled ESC-MSCs were found in the lung and skeletal muscle tissue (Fig. 7a), and we found that the majority of cells populated the lung, while a few cells populated the skeletal muscle on 1 day. We also found that the cells disappeared from skeletal muscle tissue on the third day and from lung tissue on the fifth day in our study. Western blotting demonstrated that the level of CD68 (a marker of macrophages), CD86(a marker of M1 macrophage), CD206 (a marker of M2 macrophage) increased after 1 day and peaked at 3 days, after which the expression of CD68, CD86 and CD206 gradually declined, and treatment with ESC-MSCs decreased the expression of CD86, and increased the expression of CD206 and the ratio of CD206/CD86 (M2/M1) in skeletal muscle after ACS (Fig. 7b–f). Immunofluorescence staining of macrophages on Day 3 also confirmed the decreased inflation of M1 macrophage and increased inflation of M2 macrophage in skeletal muscle (Fig. 7g). Furthermore, quantitative real-time PCR showed that ESC-MSC treatment significantly decreased the mRNA expression of the proinflammatory cytokines TNF-α and IL-6 and increased the anti-inflammatory cytokine IL-10 (Fig. 7h) 3 days after ACS.

### In vivo depletion of macrophages attenuates the therapeutic effect of ESC-MSCs

**Fig. 8:**
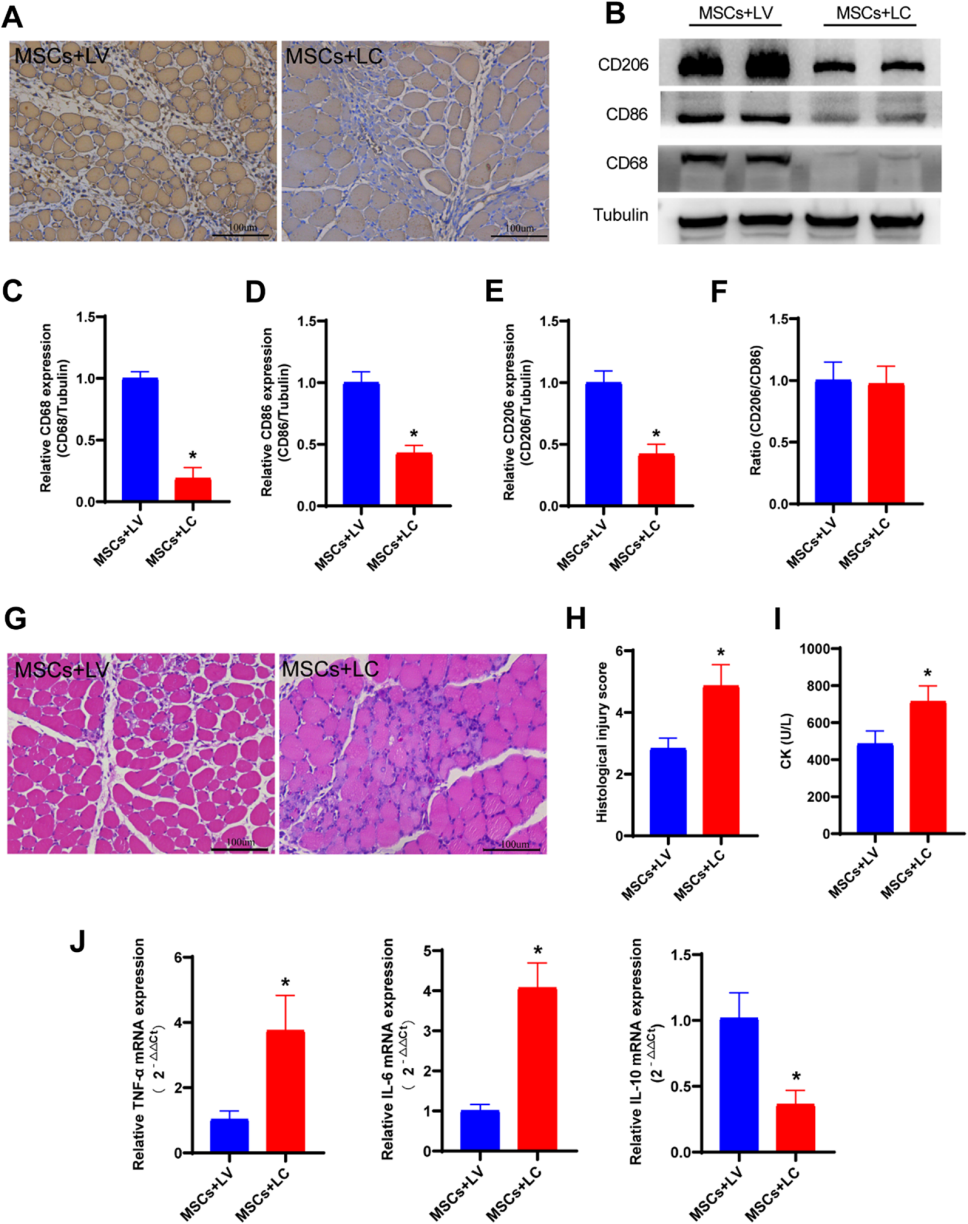
Depletion of macrophages using LC attenuated the therapeutic effect of ESC-MSCs. **a** Immunohistochemical evaluation of macrophage (CD68) infiltration in skeletal muscle after macrophage depletion. **b** Representative images showing CD68, CD86, and CD206 expression in skeletal muscle after macrophage depletion. **c** Quantitative analysis of CD68 expression. **d** Quantitative analysis of CD86 expression. **e** Quantitative analysis of CD206 expression. **f** Quantitative analysis of CD206/CD86 ratio. **g** Representative images of skeletal muscle stained with H&E after macrophage depletion. **h** Quantitative analysis of skeletal muscle injury scores after macrophage depletion. **i** Serum CK levels after macrophage depletion. **j** Quantitative real-time PCR data showing the mRNA levels of TNF-α, IL-6, and IL-10 in skeletal muscle after macrophage depletion. *N* = 7. The data are presented as the mean ± SD. *P < 0.05 versus rats injected with LV

It was determined that pro-inflammatory CD86 + (M1) macrophages were decreased and anti-inflammatory CD206 + (M2) macrophages were increased in the ESC-MSC treatment group. LC was injected into ESC-MSCs treated ACS rats to deplete macrophages, and LV was administered as a control (Fig. 1, Part 3). Immunohistochemistry showed a significant decrease in the macrophage marker CD68 in skeletal muscle tissue (Fig. 8a), and western blotting also confirmed a decrease in the macrophage-associated protein expression in skeletal muscle tissue 3 days after macrophage depletion (Fig. 8b–e). Skeletal muscle fiber injury (Fig. 8g-h) and serum CK levels (Fig. 8i) showed that the depletion of macrophages could enhance skeletal muscle injury 3 days after ACS with ESC-MSC treatment, and quantitative real-time PCR showed that the inflammatory response was further increased in skeletal muscle tissue (Fig. 8j).

## Discussion

**Fig. 9:**
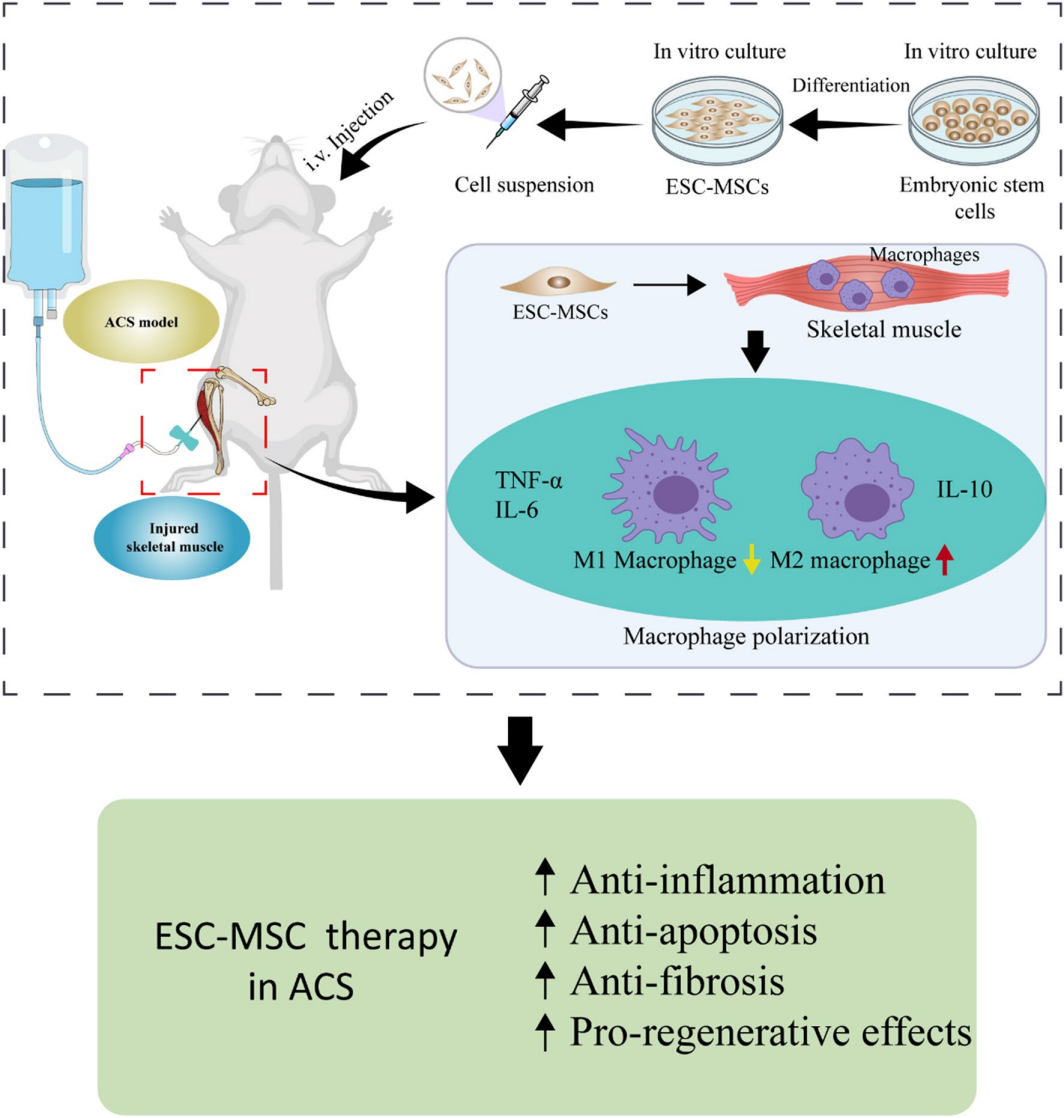
Schematic mechanism by which ESC-MSCs ameliorate skeletal muscle injury induced by ACS

In the present study, we investigate the efficacy and underlying mechanism of ESC-MSCs in ACS-induced skeletal muscle injury. It is shown that ESC-MSC therapy may serve as an effective modality for ACS treatment, and the protective effects were related to the shifting polarization of macrophages favorable to M2 other than M1 phenotype, which might mitigate inflammatory cascades and enhance subsequent reparative activities, thereby limiting reperfusion-associated skeletal muscle injury (Fig. 9). Those findings are based on the following evidence: (1) the administration of ESC-MSCs significantly alleviates ACS-induced skeletal muscle injury; (2) infused ESC-MSCs decrease M1 macrophage infiltration, increase M2 macrophage infiltration and regulate the inflammatory response in injured skeletal muscle after ACS; (3) the beneficial effects of ESC-MSCs were attenuated by the depletion of macrophages.

According to our result of serum creatine kinase, the maximum skeletal muscle cells injury was achieved on Day 1 in our study, we selected Day 1 to evaluate muscle fiber pathological injury, inflammatory cell infiltration, and systemic inflammatory response in this study. Similar to previous studies that reported beneficial effects of other MSCs on skeletal muscle injury, ESC-MSC therapy can effectively reduce the inflammatory response in vivo and inflammation-induced skeletal muscle edema 1 day after ACS, and the decrease in serum CK levels suggests that ESC-MSCs reduce skeletal muscle cell injury after ACS. Histological analysis of the TA muscle on Day 1 also confirmed that ESC-MSCs significantly reduced inflammatory cell infiltration and the disorganization of skeletal muscle fibers. Apoptosis is considered to be an inevitable type of cell death induced by IRI [36–38], especially in skeletal muscle [39, 40]. Because muscle edema began to resolve on Day 3 in our study, we evaluated the level of apoptosis in TA muscle tissue as an indicator of muscle injury after ACS. By decreasing inflammation, ESC-MSCs significantly attenuated apoptosis and regulated the expression of apoptosis-associated proteins. These findings suggest that ESC-MSCs can reduce skeletal muscle injury and subsequent apoptosis by reducing inflammation after ACS. Regeneration and fibrosis in injured skeletal muscle usually occur between 7 and 10 days after injury [31]. We chose 7 days after ACS to evaluate the effect of ESC-MSCs on the regeneration and fibrosis of skeletal muscle injury. Histological analysis showed that ESC-MSCs significantly decreased fibrosis and enhanced muscle regeneration (both myofiber numbers and diameters) 7 days after ACS injury. With the observed increase in muscle function recovery, our data suggest that ESC-MSCs can reduce fibrosis, enhance skeletal muscle regeneration, and improve functional recovery of skeletal muscle after ACS-induced injury. These data suggest that ESC-MSC therapy can ameliorate skeletal muscle injury induced by ACS, but the exact mechanism remains unknown.

Macrophages are the main source of cytokines, chemokines and growth factors that guide inflammation and repair after skeletal muscle injury [41], and exhibit two phenotypes with different functions: the classically activated subtype (M1) displays a proinflammatory profile, and the alternatively activated subtype (M2) exhibits anti-inflammatory and tissue repair properties [42]. Previous studies have shown that other sources of MSCs have beneficial effects on IRI-related injury by facilitating the transformation of M1-type macrophages to M2-type macrophages [43–45], and experiments showed that M1 macrophages co-cultured with MSCs acquired an M2 phenotype [46]. However, the role of macrophages in ESC-MSCs treated IRI-related skeletal muscle injury, such as that in ACS, remains unclear. Therefore, we labeled ESC-MSCs before injection and investigated the relationship between ESC-MSC therapy and macrophages in ACS injury. ESC-MSCs disappeared from skeletal muscle tissue on the third day, suggesting that other cells may play a role in ESC-MSC therapy.

CD68, CD86, and CD206 are cell membrane proteins for macrophages, M1 macrophages, and M2 macrophages, respectively [47]. These proteins were analyzed to reflect macrophages infiltration in skeletal muscle tissue. Western blotting indicated that ESC-MSC therapy decreased the expression of CD86 and increased the expression of CD206. Immunofluorescence staining further confirmed that M1 macrophage infiltration was decreased and M2 macrophage infiltration was increased in skeletal muscle tissue in the ESC-MSC treatment group. The mRNA expression of inflammatory factors was analyzed, and the results showed that inflammation was reduced in the cell therapy group 3 days after ACS, at which time M2/M1 ratio was highest.

To confirm the role of macrophages in ESC-MSCs mediated attenuation of ACS-induced skeletal muscle injury, we established a rat model of macrophage depletion by intravenous injection of LC as previously described [26]. Because the infiltration of macrophages was most abundant on the third day in our study, we selected 3 days after ACS as the observation point. Our results showed that LC or LV may have little effect on the polarization of macrophages in ESC-MSCs therapy, and the protective effect of ESC-MSCs on ACS-induced skeletal muscle injury was weakened after macrophage depletion, and inflammation in skeletal muscle was further increased. These observations suggest that the effects of ESC-MSCs largely relied on its interactions with macrophages.

This study was the first to demonstrate an improvement in ACS-induced skeletal muscle injury mediated by human ESC-MSCs. Our study identified a stable, reliable, and consistent source of MSCs for treating ACS or similar skeletal muscle IRI diseases in the clinic. Furthermore, our study of the roles of macrophages in ESC-MSCs mediated treatment of ACS-induced skeletal muscle injury should provide the basis for the functional studies needed to determine the efficacy of ESC-MSCs in the future.

Several limitations should not be ignored in the current study. First, a single dose of ESC-MSCs was chosen to treat the animals in this study, the optimal dosage of ESC-MSCs needs to be confirmed in the future. Second, although our study suggests that macrophages polarization plays an important role in ESC-MSC treatment, the molecular basis of ESC-MSCs regulating macrophage polarization under ACS conditions remains unknown. Third, the findings of a rodent study may not necessarily apply to humans, and macrophages may have differences between humans and rats. Therefore, further studies on clinical applications and elucidating the role of ESC-MSCs in regulating macrophage polarization would be desirable.

## Conclusions

Our findings suggest that embryonic stem cell-derived mesenchymal stem cells infusion could effectively alleviate ACS-induced skeletal muscle injury, in which the protective effects were related to the regulation of macrophage polarization.

## Data Availability

The original contributions presented in the study are included in the article. Further inquiries can be available on request to the corresponding author.

## Abbreviations

ACS: Acute compartment syndrome
IRI: Ischemia–reperfusion injury
MSCs: Mesenchymal stem cells
ESCs: Embryonic stem cells
ESC-MSCs: Embryonic stem cell-derived mesenchymal stem cells
EBs: Embryoid bodies
TA: Tibialis anterior
CK: Creatine kinase
ELISA: Enzyme-linked immunosorbent assay
TUNEL: Terminal dUTP nick end labeling
LC: Liposomal clodronate
LV: Liposomal vehicle
